# Feasibility of spectral shaping for detection and quantification of coronary calcifications in ultra-low dose CT

**DOI:** 10.1007/s00330-016-4507-z

**Published:** 2016-08-29

**Authors:** Marleen Vonder, Gert Jan Pelgrim, Sèvrin E. M. Huijsse, Mathias Meyer, Marcel J. W. Greuter, Thomas Henzler, Thomas G. Flohr, Matthijs Oudkerk, Rozemarijn Vliegenthart

**Affiliations:** 1 Department of Radiology, University of Groningen, University Medical Center Groningen, Hanzeplein 1, EB44, 9713 GZ Groningen, The Netherlands; 2 Center for Medical Imaging North-East Netherlands (CMI-NEN), University of Groningen, University Medical Center Groningen, Hanzeplein 1, Groningen, The Netherlands; 3 Institute of Clinical Radiology and Nuclear Medicine, Medical Faculty Mannheim, University Medical Center Mannheim, Heidelberg University, Heidelberg, Germany; 4 Siemens Healthcare GmbH, Computed Tomography, Forchheim, Germany

**Keywords:** Computed tomography, Coronary arteriosclerosis, Cardiovascular Diseases, Imaging, Mass screening

## Abstract

**Objectives:**

To evaluate detectability and quantification of coronary calcifications for CT with a tin filter for spectral shaping.

**Methods:**

Phantom inserts with 100 small and 9 large calcifications, and a moving artificial artery with 3 calcifications (speed 0–30 mm/s) were placed in a thorax phantom simulating different patient sizes. The phantom was scanned in high-pitch spiral mode at 100 kVp with tin filter (Sn100 kVp), and at a reference of 120 kVp, with electrocardiographic (ECG) gating. Detectability and quantification of calcifications were analyzed for standard (130 HU) and adapted thresholds.

**Results:**

Sn100 kVp yielded lower detectability of calcifications (9 % versus 12 %, *p* = 0.027) and lower Agatston scores (*p* < 0.008), irrespective of calcification, patient size and speed. Volume scores of the moving calcifications for Sn100 kVp at speed 10–30 mm/s were lower (*p* < 0.001), while mass scores were similar (*p* = 0.131). For Sn100 kVp with adapted threshold of 117 HU, detectability (*p* = 1.000) and Agatston score (*p* > 0.206) were similar to 120 kVp. Spectral shaping resulted in median dose reduction of 62.3 % (range 59.0–73.4 %).

**Conclusions:**

Coronary calcium scanning with spectral shaping yields lower detectability of calcifications and lower Agatston scores compared to 120 kVp scanning, for which a HU threshold correction should be developed.

***Key points*:**

• *Sn100kVp yields lower detectability and lower Agatston scores compared to 120kVp*

• *Adapted HU threshold for Sn100kVp provides Agatston scores comparable to 120kVp*

• *Sn100 kVp considerably reduces dose in calcium scoring versus 120 kVp*

## Introduction

Since the publication of the considerable lung cancer mortality reduction due to computed tomography (CT) screening in the National Lung Screening Trial [1], lung cancer screening has become recommended by US and European healthcare organizations [2–4]. The advent of low-dose CT lung cancer screening may also offer the opportunity to screen for coronary calcifications. It is well known that smoking, the main risk factor for lung cancer, is also a major risk factor for cardiovascular disease. Thus, screening for cardiovascular disease combined with early detection of lung cancer, may improve the cost-effectiveness of chest CT screening.

The dose of chest CT imaging in third generation dual-source CT (DSCT) can be further reduced by spectral shaping of the x-ray beam due to pre-filtration by a tin filter placed behind the x-ray tube. The potential for radiation dose reduction in lung cancer screening with this ultra-low dose chest CT was recently shown [5, 6]. A dose reduction up to 90 % was achieved with spectral shaping and advanced iterative reconstruction, while maintaining a high sensitivity of pulmonary nodule detection [5].

The use of spectral shaping by a tin filter leads to a narrower x-ray tube spectrum with fewer quanta at low energies, which shifts the mean energy of the spectrum to higher values [5]. The higher mean photon energy results in lower Hounsfield Unit (HU) values of tissues, including coronary calcifications. The maximum HU -value of a calcification affects the calcium score because of the multi-threshold calculation in the Agatston scoring method [7]. Calcifications with a maximum value below a threshold of 130 HU are not included in the Agatston score. It is unknown how the higher mean photon energy due to the tin filter influences the detectability of small coronary calcifications and the resulting calcium scores for various patient sizes and for moving coronary arteries. When changing the CT protocol for calcium scoring by spectral shaping, it is essential that the detectability of coronary calcification remains similarly high, and that the resulting calcium scores are comparable with original results in order not to affect cardiovascular risk stratification.

The purpose of this phantom study was to evaluate the detectability and quantification of coronary calcifications for a CT system with a tin filter for spectral shaping.

## Materials and methods

### Phantom

**Fig. 1:**
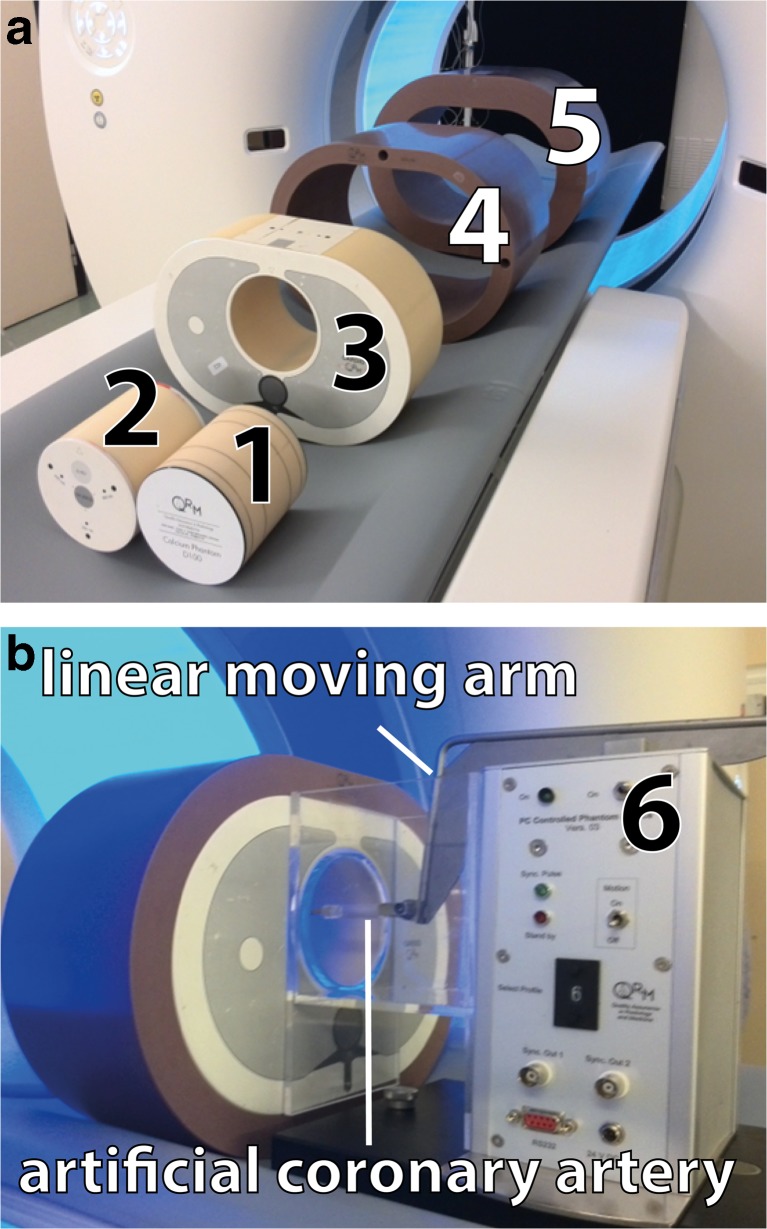
The QRM cardiac phantom set-up with different calcium inserts. **(a**, **b)** The inserts (1, 2, 6) were consecutively placed in the thorax phantom (3) and additional rings (4, 5) were used to simulate medium and large patient sizes. 1) D100 insert; 2) CCI insert; 3) Thorax phantom; 4) Phantom ring representing a medium patient size; 5) Phantom ring representing a large patient size; 6) Sim2D in which the artificial coronary artery with three calcifications is placed

An anthropomorphic chest CT phantom (Thorax, QRM, Möhrendorf, Germany) with a cylindrical opening at the position of the heart was used to simulate a small patient size. Fat-equivalent extension rings (extension rings M and L, QRM, Möhrendorf) were placed around the phantom to mimic medium and large patient size (see Fig. 1).

Three inserts with calcifications mimicking coronary calcium were consecutively placed into the cylindrical opening of the phantom. The first insert, D100, contained 100 small cylindrical calcifications (D100, QRM, Möhrendorf) varying in density (90–540 mgHA/cm^3^) and size (0.1–6.3 mm^3^) [8]. The D100 insert was used to determine the detectability of small calcifications. The second insert, CCI, contained 9 slightly larger cylindrical calcifications (CCI, QRM, Möhrendorf) with varying density (200, 400 and 800 mgHA/cm^3^) and size (0.8, 21.2 and 98.2 mm^3^) [9]. The CCI insert was used to determine the Agatston score, volume and mass for larger calcifications. The third insert consisted of a linear moving artificial coronary artery (Sim2D, QRM, Möhrendorf, Germany) with 3 calcified lesions, with density of 401 mgHA/cm^3^ and varying size (9.1, 24.6 and 62.8 mm^3^) [10]. The coronary artery moved at a speed of 0, 10, 20 and 30 mm/s, corresponding to the expected velocity range of an in -vivo coronary artery during ECG- gated acquisitions [10, 11].

### Image acquisition

The thorax phantom and calcium inserts were scanned with a third- generation DSCT scanner (Somatom Force, Siemens, Erlangen, Germany) in high-pitch spiral mode. As a reference, data sets were acquired at a tube voltage of 120 kVp and tube current of 90 reference mAs. Next, data sets were acquired at a tube voltage of 100 kVp with spectral shaping due to the 0.6- mm tin filter (Sn100 kVp) and tube current of 180 reference mAs. ECG gating was used for acquisitions of the moving coronary artery at Sn100 kVp and 120 kVp. The data were reconstructed with a field- of- view (FOV) of 250 mm and a 512 × 512 matrix, a sharp reconstruction filter (Qr36d) and filtered back projection at a slice thickness of 3.0 mm and an increment of 1.5 mm (to avoid over- or undersampling) [12]. Acquisitions were repeated four times for D100, five times for CCI and three times for Sim2D with a small translation and rotation between each acquisition.

### Image analysis

Calcium detectability was determined automatically using a Matlab script [8]. Analysis of the CCI and Sim2D data were performed manually with Aquarius iNtuition Viewer (Version 4.1.11, TeraRecon Inc, Foster City, CA, USA). A calcification was defined as a region with a peak value of ≥130 HU (standard calcium threshold) and comprising ≥2 adjacent voxels (eight-connected connectivity [13]). The calcium detectability was defined as the total number of detected calcifications in the D100 insert. In general, the less sensitive an acquisition is for small and low- density calcifications, the lower the number of detected calcifications [8]. For Sn100 kVp and 120 kVp, the standard calcium threshold for Agatston score, volume and mass was used, and median and interquartile ranges (IQRs) of the repetitions were calculated, and coefficient of variation for all scores were calculated [9].

In addition, optimization of detectability and calcium scores was performed by adapting the calcium threshold for Sn100 kVp. The mean HU of the four calibration planes in the D100 insert for both protocols was determined (including all repetitions and patient sizes) and the threshold for Sn100 kVp (t_Sn100 kVp_) scans was calculated, using the following formula [14]:

$$ {t}_{Sn100\kern0.5em kVp}=130\kern0.5em HU\left(\frac{mean\kern0.5em H{U}_{caHA@Sn100kVp}}{mean\kern0.5em H{U}_{caHA@Sn120kVp}}\right) $$

### Radiation dose

The radiation dose parameters, computed tomography dose index (CTDI_vol_) and dose-length product (DLP), were taken from the electronically logged protocol from each CT study.

### Statistical analysis

Kruskal–Wallis testing was used to analyze differences in detectability of calcifications, Agatston score, volume score and mass scores between Sn100 kVp and 120 kVp acquisitions. The Kruskal–Wallis test was performed separately for each patient size. If the Kruskal–Wallis test showed a significant difference in distribution of outcomes, one-by-one comparison was performed by using the Mann–Whitney U test, again for each patient size separately. Kendall’s tau-b was used to analyze trends in Agatston, volume and mass score based on speed (SPSS Statistics, version 22, IBM, USA). *P* < 0.05 was considered to indicate a statistically significant difference.

## Results

### Calcium detectability at standard calcium threshold

The overall median detectability was 9.0 (IQR: 8.0–9.9) out of 100 calcifications at Sn100 kVp, and 12.0 (IQR: 11.0–13.0) at 120 kVp (see Table 1). The overall median calcium detectability was 25 %lower at Sn100 kVp compared to the detectability at 120 kVp (*p* = 0.027).

**Table 1:** Calcium detectability of small calcifications out of a total of 100 calcifications for small, medium, and large patient size at standard calcium threshold

	Scan protocol		
	Sn100 kVp	120 kVp	Percentage difference of medians
Patient size	Median (IQR) n = *	Median (IQR) n = *	
Small	9.0 (9.0–11.3)	12.5 (12.0–13.0)	28 %
Medium	8.5 (6.5–10.5)	11.0 (11.0–12.5)	23 %
Large	8.8 (6.5–9.9)	11.5 (11.0–12.8)	25 %
All sizes	9.0 (8.0–9.9)	12.0 (11.0–13.0)	25 %

* Median number of absolute detected calcifications in four consecutive measurements

### Quantification of calcifications at standard calcium threshold

Coefficient of variation for all patient sizes at Sn100 kVp (CCI) was of comparable magnitude for the mass score (2.6 %) and Agatston score (2.7 %) and largest for the volume score (4.7 %).

**Fig. 2:**
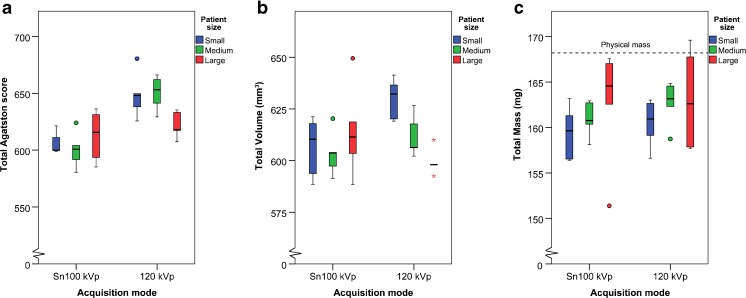
Calcium scores of three patient sizes at Sn100 kVp and 120 kVp of the CCI insert, at standard calcium threshold (130 HU). The median and interquartile ranges for: **(a)** Agatston score, **(b)** Volume, **(c)** Mass
  The Agatston score decreased significantly with increasing speed for Sn100 kVp (τ_b_ = -0.383, *p* = 0.003) and for 120 kVp (τ_b_ = -0.442, *p* = 0.001). The median Agatston score was 113.6 (IQR: 98.9–129.6) at Sn100 kVp and 149.0 (IQR: 130.0–160.0) at 120 kVp. The Agatston score was lower at Sn100 kVp compared to 120 kVp for the moving artificial coronary artery (speeds 0–30 mm/s; *p* < 0.001; see Fig. 3a).

1. Agatston score
  The overall median Agatston score at Sn100 kVp was 600.8 (IQR: 593.7–621.3) and 638.3 (IQR: 625.7–653.2) at 120 kVp for the CCI insert. The Agatston score was significantly lower at Sn100 kVp (*p* = 0.008) for each patient size, except for the CCI insert for large size (*p* = 0.548; see Fig. 2a).

**Fig. 3:**
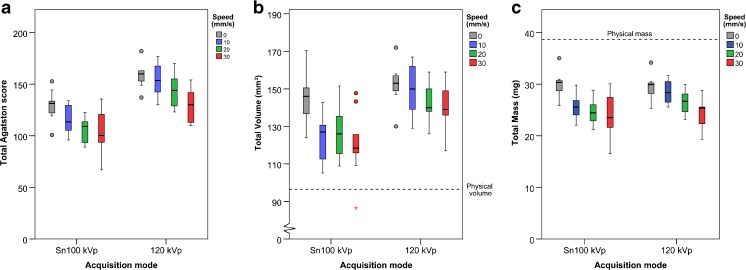
Calcium scores of the artificial coronary artery moving at speeds of 0–30 mm/s at Sn100 kVp and 120 kVp for electrocardiographically (ECG)-gated acquisitions, at standard calcium threshold (130 HU). The median and interquartile ranges for: **(a)** Agatston score, **(b)** Volume, **(c)** Mass
2. Volume score
  The median overall volume score of the CCI insert was 603.9 (IQR: 593.8–618.7) mm^3^ at Sn 100 kVp, and was 610.0 (IQR: 598.1–626.6) mm^3^ at 120 kVp, while the physical volume was 360.6 mm^3^. No significant difference in total volume score was found between Sn100 kVp and 120 kVp (*p* < 0.206) for the CCI insert (see Fig. 2b).
  The total physical volume of the calcifications in the artificial coronary artery of the Sim2D insert was 96.5 mm^3^. The median volume score was 127.9 (IQR: 116.0–143.1) mm^3^ at Sn100 kVp and 147.0 (IQR: 138.0–157.0) mm^3^ at 120 kVp (0–30 mm/s). No difference in volume score was found between Sn100 kVp and 120 kVp for measurements with no movement of the coronary artery (speed 0 mm/s; *p* = 0.347). However, the volume score was significantly lower at Sn100 kVp compared to 120 kVp taken all the measurements with the coronary artery moving at 10–30 mm/s together (*p* < 0.001). The volume score decreased significantly with increasing speed for Sn100 kVp (τ_b_ = -0.325, *p* < 0.011) as well as for 120 kVp (τ_b_ = -0.303, *p* = 0.021; see Fig. 3b).
3. Mass score
  The median overall mass score of the CCI insert at Sn100 kVp was 161.3 (IQR: 158.1–163.2) mg, and 162.6 (IQR: 158.7–164.5) mg at 120 kVp, while the physical mass was 168.2 mg. No significant difference in mass score was found between Sn100 kVp and 120 kVp (*p* < 1.00) for the CCI insert (see Fig. 2c).
  The physical mass of the calcifications in the artificial coronary artery of the Sim2D insert was 38.7 mg. The median mass score at Sn100 kVp was 25.9 (IQR: 23.0–29.1) mg and 27.1 (IQR: 25.4–29.7) mg at 120 kVp. The mass score decreased significantly with increasing speed for Sn100 kVp (τ_b_ = -0.459, *p* < 0.001) as well as for 120 kVp (τ_b_ = -0.507, *p* < 0.001; see Fig. 3c). No significant difference in mass score was found for Sn100 kVp and 120 kVp for measurements with no (speed 0 mm/s, *p* = 0.347) or minor movement (10–30 mm/s, *p* = 0.131) of the coronary artery.

### Optimization of detectability and calcium scores for Sn100 kVp

The adapted calcium threshold for Sn100 kVp was 117 HU. The overall median detectability at Sn100 kVp with calcium threshold of 117 HU was 11.5 (IQR: 10.0–12.8) out of 100 calcifications, and no significant difference in detectability was found compared to 120 kVp (*p* = 1.000).

The overall median Agatston score of the CCI insert was 657.4 (IQR: 651.1–-675.2). No significant difference in median Agatston score was found at Sn100 kVp with 117 HU threshold compared to 120 kVp for small and medium patient size (*p* > 0.206). However, at Sn100 kVp and 117 HU the volume (*p* < 0.008) and mass (*p* < 0.008) score of the CCI insert were significantly higher for small and medium patient size compared to 120 kVp.

### Radiation dose

The mean CTDI_vol_ for small to large patient size was 0.16–0.65 mGy at Sn100 kVp and 0.44–2.44 mGy at 120 kVp. The mean DLP was 1.6–6.5 mGy∙cm and 4.3–24.4 mGy∙cm for Sn100 kVp and 120 kVp, respectively.

## Discussion

This phantom study evaluated the detectability and quantification of coronary calcifications using third- generation DSCT with spectral shaping, by comparing a Sn100 kVp protocol with the reference 120 kVp scan protocol. We found that the Sn100 kVp scan protocol with standard HU threshold, resulted in lower detectability of calcifications and lower calcium scores regardless of coronary movement. Adaptation of the HU threshold for calcium scoring at Sn100 kVp resulted in similar detectability and Agatston score for small and medium patient size. Besides, the Sn100 kVp protocol led to a major decrease in radiation dose of 63 % to 73 % for, respectively, small to large patient size.

Until now, the use of spectral shaping in CT scanning has only been examined for non-contrast enhanced chest CT [5, 6] and recently in one cardiac CT study by McQuiston et al. in a simplified setting [15]. In this study, we comprehensively describe the impact of a tin filter in coronary calcification scanning. The 120- kVp protocol at third- generation DSCT showed calcium score results similar to those obtained on previous CT systems [9, 16, 17]. For example, the mean Agatston score of the CCI insert at 120 kVp was 640.4, which falls within the range of the Agatston score of 605.0–655.4 based on different MDCTs in the study of McCollough et al. [9]. The Agatston score for the CCI insert was slightly lower for the Sn100 kVp protocol compared to the scores determined with general coronary calcium protocols on various CT systems [9, 16, 17]. For example, the mean Agatston score for Sn100 kVp was 606.3, which is at the lower limit of the range reported by McCollough et al. [9]. The study by McQuistion et al. recently showed no significant difference in Agatston score between Sn100 kVp and 120 kVp for the CCI insert for small patient size using standard HU threshold [15], while our study did show a difference in Agatston score for small patient size. Possibly, the use of various tube currents and lack of scan repetition in the study of McQuistion et al. explains this difference. Furthermore, the studies of Ulzheimer et al. and Groen et al. showed that calcium scores decrease with increasing heart rate, which is in accordance with our results for increasing speed of the moving artificial coronary artery for 120 kVp as well as for Sn100 kVp [18, 19]. Based on these findings, we assume that the 120- kVp protocol on third- generation DSCT leads to results similar to those from coronary calcium scan protocols on other CT- systems, while the Sn100 kVp protocol leads to lower calcium scores compared to the protocols on other CT- systems. On the contrary, McQuiston et al. suggested that use of a tin filter does not impact the calcium score [15]. However, in their study, small calcifications (sub-millimeter), different patient sizes, coronary artery movement, calcium mass and inter-scan variability were not included, while all these variables by themselves are important factors to consider in calcium scoring.

The results of our study imply that the use of the Sn100- kVp protocol for coronary calcium scanning could have two major consequences in determining the appropriate cardiovascular risk in clinical practice. Firstly, the lower calcification detectability of the Sn100- kVp protocol at standard threshold of 130 HU could lead to underestimation and false-negative calcium scores in patients. This would result in addressing a patient’s cardiovascular risk inappropriately as ‘very low’. Secondly, the use of spectral shaping led to lower Agatston scores, which may result in reclassification of the patient into a lower cardiovascular risk category. Hence, a lower Agatston score or false-negative score could lead to an underestimation of the cardiovascular risk. In this study we tried to compensate for this underestimation of Sn100 kVp by lowering the calcium threshold for Sn100 kVp from 130 HU to 117 HU. This improved the detectability and Agatston score for Sn100 kVp, such that there was no longer a significant difference with conventional 120 kVp. However, the volume and mass score were higher at Sn100 kVp compared to 120kVp, but these scores, although more reproducible, are not used in clinical practice.

The large reduction in radiation dose with the Sn100- kVp protocol is a large advantage over the conventional 120- kVp protocol. This is of special interest when screening of coronary calcium is combined with lung cancer screening or in the case that population screening for coronary calcium is introduced in clinical practice. This study does not provide answers with regard to possible reclassification of screenees if a Sn100- kVp protocol is used. This should be investigated in future studies. Besides, a recent study showed that ungated chest CT acquisitions might be used for CAC risk stratification [20], simplifying a combined coronary calcium and lung screening acquisition.

This study has some limitations. First, the tube current was modulated for each patient size. However, for the large patient size at Sn100 kVp, the tube current could not be further increased beyond a quality reference of 180 mAs, in case of the high-pitch spiral mode. This resulted in a higher noise level for the larger patient sizes at the Sn100- kVp protocol compared to the 120- kVp protocol. However, former studies have shown minor influence of tube current or noise level on coronary calcium scoring [21, 22].

A second , general, limitation is that cardiovascular risk stratification can only be performed based on the Agatston score, because of its well-known prognostic value [7, 23–28]. As an alternative to the Agatston score, several studies recommend determining the total mass of all calcifications because of its higher inter- and intra-scanner reproducibility [9, 29, 30]. Also, our study showed that the mass score is the most consistent score across patient sizes for the Sn100- kVp protocol. At the standard calcium threshold of 130 HU, in contrast to the Agatston score, no significant difference in mass score was found between Sn100 kVp and 120 kVp for any patient size for the moving coronary artery. However, with the adapted HU threshold for calcium scoring, we also found similar detectability and Agatston scores.

In conclusion, this phantom study showed that spectral shaping in coronary calcium scanning reduces the radiation dose but leads to a lower detection of calcifications and lower Agatston scores, which could result in underestimation of the cardiovascular risk in patients. In the future, underestimation may be corrected by adapting the HU threshold for calcium scoring using a scan protocol with spectral shaping.

## Abbreviations

CT: Computed tomography
ECG: Electrocardiographic
DSCT: Dual source computed tomography
HU: Hounsfield unit
Sn100 kVp: Tube voltage of 100 kVp with tin spectral shaping
FOV: Field -of -view
CTDIvol: Computed tomography dose index
DLP: Dose-length product
IQR: Interquartile range

## References

[1] The National Lung Screening Trial Research Team (2015). Reduced Lung-Cancer Mortality with Low-Dose Computed Tomographic Screening. N Engl J Med.

[2] Wender R, Fontham ETH, Barrera E (2013). American Cancer Society Lung Cancer Screening Guidelines Richard. CA Cancer J Clin.

[3] Jaklitsch MT, Jacobson FL, Austin JHM (2012). The American Association for Thoracic Surgery guidelines for lung cancer screening using low-dose computed tomography scans for lung cancer survivors and other high-risk groups. J Thorac Cardiovasc Surg.

[4] Kauczor H-U, Bonomo L, Gaga M (2015). ESR/ERS white paper on lung cancer screening. Eur Radiol.

[5] Gordic S, Morsbach F, Schmidt B (2014). Ultralow-Dose Chest Computed Tomography for Pulmonary Nodule Detection. Investig Radiol.

[6] Newell JD, Fuld MK, Allmendinger T (2015). Very Low-Dose (0.15 mGy ) Chest CT Protocols Using the COPDGene 2 Test Object and a Third-Generation Dual-Source CT Scanner With Corresponding Third-Generation Iterative Reconstruction Software. Investig Radiol.

[7] Agatston AS, Janowitz FWR, Hildner FJ (1990). Quantification of Coronary Artery Calcium Using Ultrafast Computed Tomography. JACC.

[8] Groen JM, Kofoed KF, Zacho M (2013). Calcium score of small coronary calcifications on multidetector computed tomography: Results from a static phantom study. Eur J Radiol.

[9] McCollough CH, Ulzheimer S, Halliburton SS (2007). Coronary Artery Calcium: A Multi-institutional, Multimanufacturer International Standard for Quantification at Cardiac CT. Radiology.

[10] Xie X, Greuter MJW, Groen JM (2013). Can nontriggered thoracic CT be used for coronary artery calcium scoring? A phantom study. Med Phys.

[11] Husmann L, Leschka S, Frauenfelder T (2007). Coronary Artery Motion and Cardiac Phases: Dependency on Heart Rate - Implication for CT Image Reconstruction. Radiology.

[12] Weininger M, Ritz KS, Schoepf UJ (2012). Interplatform Reproducibility of CT Coronary Calcium Scoring Software. Radiology.

[13] van Ooijen PMA, Vliegenthart R, Witteman JC, Oudkerk M (2005). Influence of scoring parameter settings on Agatston and volume scores for coronary calcification. Eur Radiol.

[14] Thomas CK, Mühlenbruch G, Wildberger JE (2006). Coronary artery calcium scoring with multislice computed tomography: in vitro assessment of a low tube voltage protocol. Investig Radiol.

[15] McQuiston AD, Muscogiuri G, Schoepf UJ (2016). Approaches to ultra-low radiation dose coronary artery calcium scoring based on 3rd generation dual-source CT: A phantom study. Eur J Radiol.

[16] Deprez FC, Vlassenbroek A, Raaijmakers R, Coche E (2013). Controversies about effects of low-kilovoltage MDCT acquisition on Agatston calcium scoring. J Cardiovasc Comput Tomogr.

[17] Fujioka C, Funama Y, Kiguchi M (2012). Coronary Artery Calcium Scoring on Different 64-detector Scanners Using a Low-tub Voltage (80kVp). Acad Radiol.

[18] Ulzheimer S, Kalender WA (2003). Assessment of calcium scoring performance in cardiac computed tomography. Eur Radiol.

[19] Groen JM, Greuter MJW, Vliegenthart R (2008). Calcium scoring using 64-slice MDCT, dual source CT and EBT: a comparative phantom study. Int J Cardiovasc Imaging.

[20] Hutt A, Duhamel A, Deken V (2016). Coronary calcium screening with dual-source CT: reliability of ungated, high-pitch chest CT in comparison with dedicated calcium-scoring CT. Eur Radiol.

[21] Shemesh J, Evron R, Koren-Morag N (2005). Coronary artery calcium measurement with multi-detector row CT and low radiation dose: comparison between 55 and 165 mAs. Radiology.

[22] Takahashi N, Bae KT (2003). Quantification of coronary artery calcium with multi-detector row CT: assessing interscan variability with different tube currents pilot study. Radiology.

[23] Rumberger JA, Simons DB, Fitzpatrick LA (1995). Coronary Artery Calcium Area by Electron-Beam Computed Tomography and Coronary Atherosclerotic Plaque Area - A Histopathologic Correlative Study. Circulation.

[24] Becker CR, Knez A, Jakobs TF (1999). Detection and quantification of coronary artery calcification with electron-beam and conventional CT. Eur Radiol.

[25] Hoff JA, Chomka EV, Krainik AJ (2001). Age and Gender Distributions of Coronary Artery Calcium Detected by Electron Beam Tomography in 35,246 Adults. Am J Cardiol.

[26] McClelland RL, Chung H, Detrano R (2006). Distribution of Coronary Artery Calcium by Race, Gender, and Age: Results from the Multi-Ethnic Study of Atherosclerosis (MESA). Circulation.

[27] Hoff JA, Daviglus ML, Chomka EVAV (2003). Conventional Coronary Artery Disease Risk Factors and Coronary Artery Calcium Detected by Electron Beam Tomography in 30,908 Healthy Individuals. Ann Epidemiol.

[28] Nucifora G, Bax JJ, Van Werkhoven JM (2011). Coronary artery calcium scoring in cardiovascular risk assessment. Cardiovasc Ther.

[29] Detrano R, Tang W, Kang X (1995). Accurate coronary calcium phosphate mass measurements from electron beam computed tomograms. Am J Card Imaging.

[30] Dijkstra H, Greuter MJW, Groen JM (2010). Coronary calcium mass scores measured by identical 64-slice MDCT scanners are comparable: a cardiac phantom study. Int J Cardiovasc Imaging.

